# Inflated prediction accuracy of neuropsychiatric biomarkers caused by data leakage in feature selection

**DOI:** 10.1038/s41598-021-87157-3

**Published:** 2021-04-12

**Authors:** Miseon Shim, Seung-Hwan Lee, Han-Jeong Hwang

**Affiliations:** 1 Department of Electronics and Information, Korea University, 2511, Sejong-ro, Jochiwon-eup, Sejong-si, 30019 Republic of Korea; 2 Psychiatry Department, Ilsan Paik Hospital, Inje University, Goyang, Republic of Korea; 3 Clinical Emotion and Cognition Research Laboratory, Goyang, Republic of Korea; 4 Interdisciplinary Graduate Program for Artificial Intelligence Smart Convergence Technology, Korea University, Sejong, 30019 Republic of Korea

**Keywords:** Psychiatric disorders, Biomarkers, Diagnostic markers, Predictive markers, Prognostic markers

## Abstract

In recent years, machine learning techniques have been frequently applied to uncovering neuropsychiatric biomarkers with the aim of accurately diagnosing neuropsychiatric diseases and predicting treatment prognosis. However, many studies did not perform cross validation (CV) when using machine learning techniques, or others performed CV in an incorrect manner, leading to significantly biased results due to overfitting problem. The aim of this study is to investigate the impact of CV on the prediction performance of neuropsychiatric biomarkers, in particular, for feature selection performed with high-dimensional features. To this end, we evaluated prediction performances using both simulation data and actual electroencephalography (EEG) data. The overall prediction accuracies of the feature selection method performed outside of CV were considerably higher than those of the feature selection method performed within CV for both the simulation and actual EEG data. The differences between the prediction accuracies of the two feature selection approaches can be thought of as the amount of overfitting due to selection bias. Our results indicate the importance of correctly using CV to avoid biased results of prediction performance of neuropsychiatric biomarkers.

## Introduction

Machine learning (ML) has attracted increasing interest in the development of neuropsychiatric biomarkers^1,2^. However, recent review and meta-analysis articles revealed that many studies did not perform cross validation (CV) when utilizing ML techniques to exploit neuropsychiatric biomarkers^1,3^, which significantly deteriorates the generalizability of neuropsychiatric biomarkers due to the overfitting problem^1^. CV is a way to estimate the performance of a prediction model^4^, where some of given data (training data) are used to train a prediction model and the others (test data) are used to estimate the performance of the model. The most important point when applying CV is that the training data must be completely separated from the test data to avoid the overfitting of a prediction model^5,6^. Otherwise, the prediction performance estimated is inflated, resulting in biased results.

A neuroimaging modality used to study neuropsychiatric biomarkers, such as electroencephalography (EEG)^7–10^, provides high-dimensional spectral-spatiotemporal features, and thus feature selection is generally performed to remove redundant features and find the optimal feature subset for accurate prediction. Feature selection should be performed within CV on each training data set independently to prevent selection bias^11,12^; features selected using the training data in each CV loop are used to build a prediction model and the corresponding features in the test data are used to evaluate the performance of the prediction model. However, there is still a recent study that feature selection was carried out on all data after which CV was performed on selected fixed features to estimate the prediction accuracy, which generally leads to selection bias as well as a biased prediction accuracy^11,12^. The objective of this study is to investigate the effect of the feature selection independently performed outside of CV on the prediction performance, thereby openly debating relevant issues for future studies. To this end, we applied two different feature selection strategies performed outside of CV and within CV for both simulation data and actual clinical EEG data, respectively, and compared the classification performance of the two different feature selection strategies. Furthermore, we investigated to what extend the features selected by the two different feature selection strategies were different, and interpreted the result from a neurophysiological point of view.

## Methods

In the recent study^13^ that used ML for predicting prognosis of depression with escitalopram treatment from EEGs, 6,424 EEG features were extracted for responders and non-responders to escitalopram treatment, and feature selection was performed with a repeated random sub-sampling method on all samples of the data. 80% of samples were randomly selected from each class and an unpaired t-test was performed for each feature, which was repeated 100 times. If a p-value was less than a significant level (< 0.05), one vote was counted for a corresponding feature, otherwise 0 vote. Five different feature sets were selected based on five different vote thresholds (≥ 50, 60, 70, 80, and 90), respectively, after which a support vector machine (SVM) classifier with a 10-fold CV was used to estimate the prediction performance. Note that even though a random sub-sampling method was used, the feature selection was ultimately performed on all samples outside of CV, and thus the each of the five feature sets fixed after the feature selection step was used during the CV to estimate the prediction performance.

**Figure 1:**
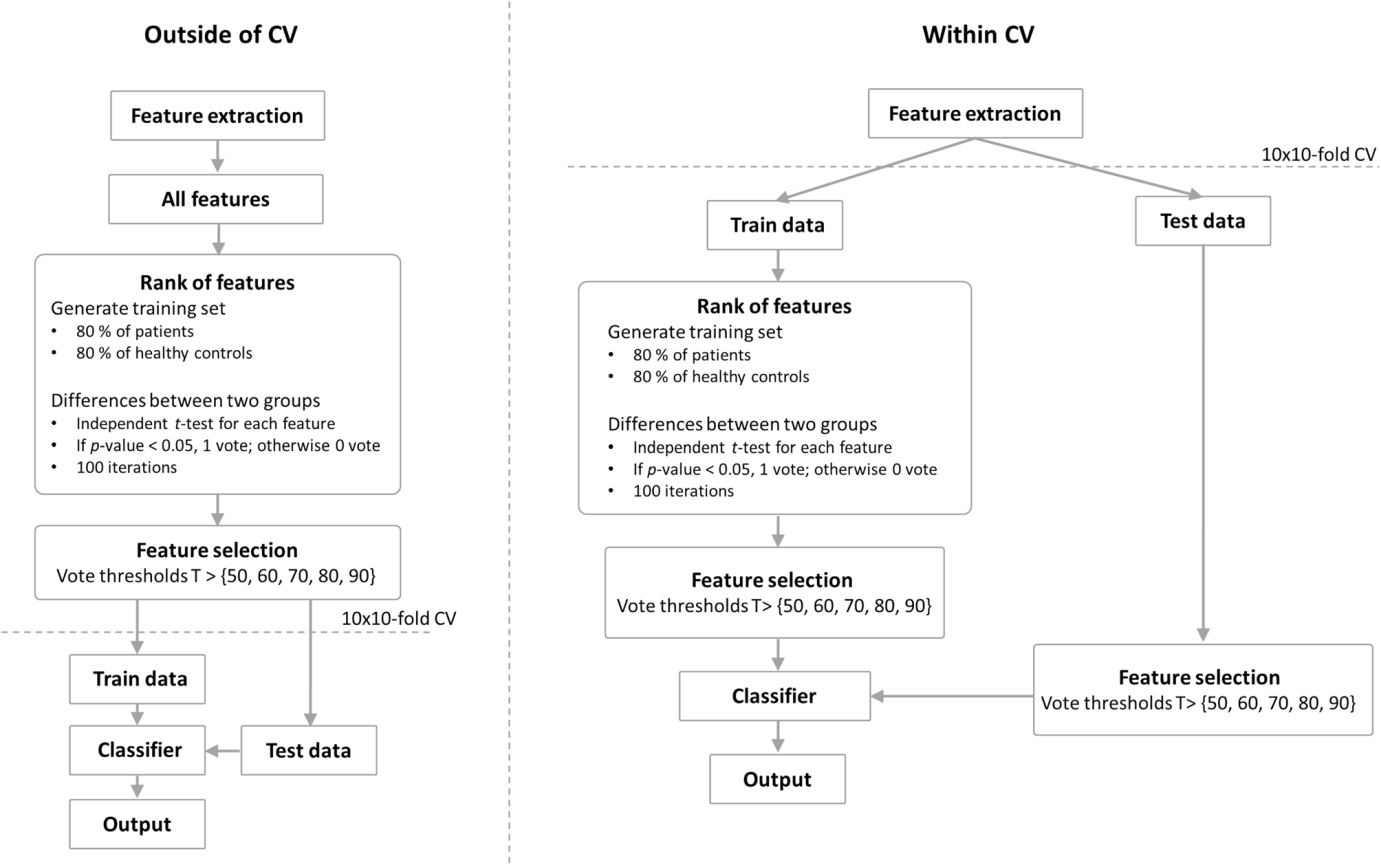
Flowchart of two different feature selection strategies. Left panel: all features were used for the feature selection method performed outside of CV, and then selected fixed features were used to evaluate the prediction performance, at which time CV was applied. Right panel: contrary to the feature selection method performed outside of CV, CV was applied in the stage of feature selection for the feature selection method performed within CV, where the features in the training set were only used for feature selection, and then the corresponding features in the test set were used to evaluate the prediction performance.

To investigate the impact of the feature selection method mentioned above on the prediction performance, we first performed a simulation test using 6,424 random variable features created with the Gaussian distribution (mean 0–0.3 with an interval of 0.1; variance: 0.1–2.3 with an interval of 0.1) for 2-class prediction. The mean and variance ranges of simulation features were selected to similarly match the number of features selected for each vote threshold in the previous study via trials and errors. For each feature, a pair of mean and variance was randomly selected within each of the mean and variance ranges to generate a feature vector, and this procedure was repeated 6,424 times. The simulation features were standardized using z-score method to accurately use the linear classifiers^14–16^. Both the distributions of the generated features of each class and Matlab code used to generate the simulation data are provided in the following website: https://github.com/miseonshim/JAMAfeatures.git. We first estimated the prediction performance using the same approach used in the previous study^13^, except that a 10 × 10-fold CV was performed and another classifier (regularized linear discriminant analysis; RLDA) was additionally introduced to increase the generalization of our study. We also estimated the prediction performance using the same approach to the previous method^13^, but at which time feature selection was performed within CV, and thus different features were selected in each CV loop. That is, features were selected in each CV loop using only training data based on the vote threshold approach, separating the test data completely from the training data, and then prediction accuracy was estimated using corresponding features in the test data. The classification accuracies were independently estimated using the five different feature sets selected by the different vote thresholds (≥ 50, 60, 70, 80, and 90), respectively. Note that we tested two other feature selection methods, reduced SVM (RSVM) and SVM-recursive feature elimination (SVM-REF), but we will only report the results of the t-test-based vote threshold approach due to its higher performance as compared to RSVM and SVM-RFE. Figure 1 represents the flowchart of the two different feature selection strategies performed outside of CV and within CV used in this study.

In addition to the simulation study, we performed the same analysis using actual clinical EEG data to check whether the results of the simulation study are transferred to real EEG data. To this end, we used resting-state EEG data recorded from 58 post-traumatic stress disorder (PTSD) patients and 58 healthy controls. The study protocol was approved by the Institutional Review Board of Inje University Ilsan Paik Hosipital [IRB number: 2015-09-018]. This study was performed in accordance with approved guidelines, and all participants provided written informed consent. The EEG data were recorded for 5 min. with eyes closed at a sampling rate of 1,000 Hz using 64 Ag/AgCl electrodes evenly mounted on the scalp according to the extended international 10–20 system (NeuroScan SynAmps2 (Compumedics USA, El Paso, TX, USA); references: M1 and M2). Eye movement artifacts were removed using established mathematical procedures based on regression approach^14^, and other gross artifacts were rejected by visual inspection. After removing the artifacts, artifact-free EEG data were used for computing features. Since altered functional connectivity was regarded as a distinct neurophysiological biomarker of PTSD patients, functional connectivity was used as the feature to evaluate the classification performance of PTSD patients and healthy controls. Among various functional connectivity indices, phase locking value (PLV) was calculated between all possible pairs of channels for six different frequency bands (delta [1–4 Hz], theta [4–8 Hz], alpha [8–12 Hz], low-beta [12–22 Hz], high-beta [22–30 Hz], and gamma [30–55 Hz]). To generate a comparable number of features to the simulation data, uniformly selected 45 channels were used for computing PLVs (excluded channels: FPz, AF3, AF4, FC5, FC1, FC2, FC6, CP5, CP1, CP2, CP6, PO7, PO3, PO4, PO8, CB1, and CB2). Thus, a total of 5,940 PLV values (_45_C_2_ $\times$ 6 frequency bands) were extracted for each subject. The classification accuracies were evaluated for each of the feature selection methods performed outside of CV and within CV, respectively, as same for the simulation data. Furthermore, we compared the spatial distributions of features (functional connectivity) selected by the two feature selection strategies (outside of CV vs. within CV) to investigate to what extend they were different. To quantify the difference, we counted the number of overlap features and divided it from the total number of features selected by the within CV approach, which was repeated 100 times (10 × 10 CV), and the overlap ratios were averaged. The mentioned analysis was performed independently for the five vote thresholds (50, 60, 70, 80, and 90), but we will only report the result of a vote threshold of 90 because the results of the other vote thresholds showed a similar tendency in terms of both the overlap ratio and spatial distribution of functional connectivity.

## Results and interpretation

### Prediction performance for simulation data

For the feature selection method performed outside of CV, the prediction accuracy was around 80%, regardless of the number of selected features (vote thresholds), with a small variance, whereas it continuously decreased as the number of selected features increased (as vote threshold got lower) for the feature selection method performed within CV (Table 1). This trend was similarly shown for the two different classifiers. Note that the number of features selected by the within CV method was continuously changed in each CV loop due to the changes in training data, and thus we report the ranges of the number of features selected during CV for the feature selection method performed within CV.

**Table 1:** Prediction accuracies of the simulation data.

Vote threshold	Outside of CV			Within CV		
	n	SVM	RLDA	n	SVM	RLDA
≥ 50	44	83.03	84.64	42–61	50.17	50.50
≥ 60	31	81.11	83.42	26–42	55.75	57.25
≥ 70	21	80.10	82.24	20–33	58.00	61.00
≥ 80	17	79.90	81.28	13–22	63.25	68.42
≥ 90	12	78.31	78.98	8–16	68.33	71.17
Mean (S.D.)		80.49 (1.56)	82.11 (1.93)		59.10 (6.24)	61.67 (7.49)

Two feature selection approaches (outside of CV and within CV) were tested using two different classifiers (SVM and RLDA, unit: %) with respect to the different vote threshold (number of selected features).

n* number of selected features, *S.D.* standard deviation, *SVM* support vector machine, *RLDA* regularized linear discriminant analysis.

**Figure 2:**
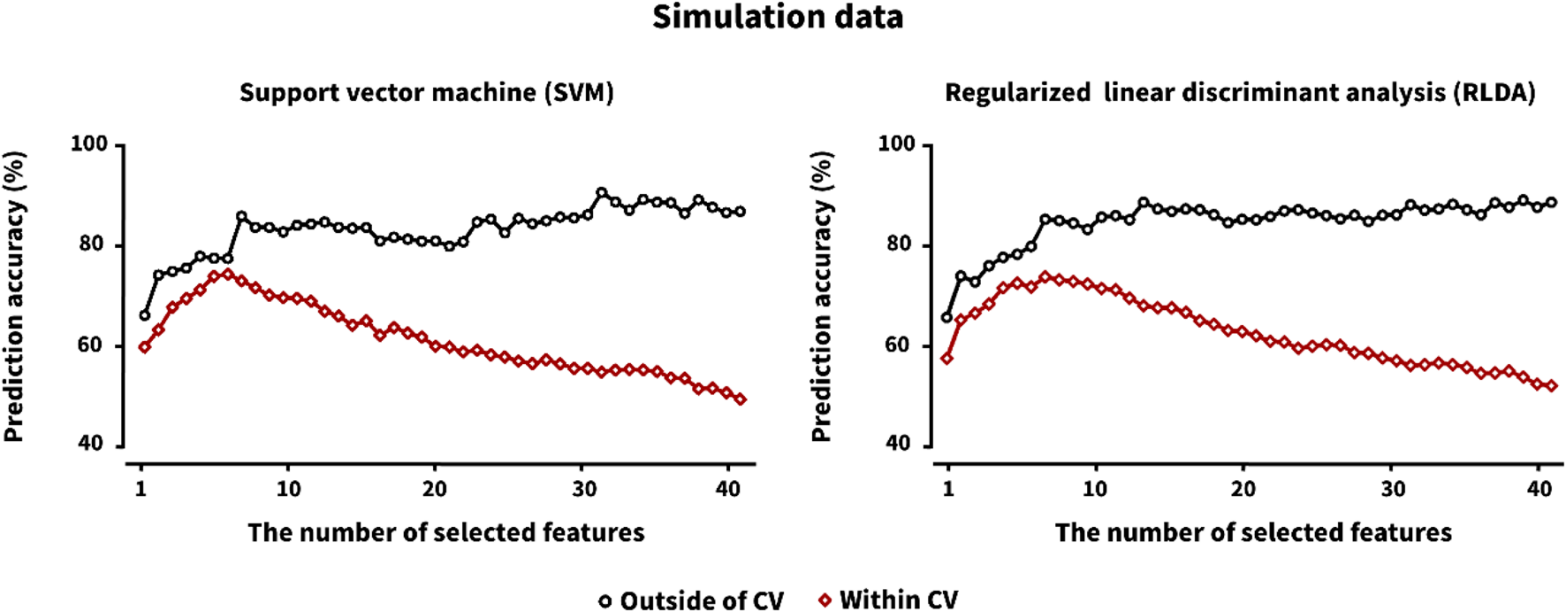
Prediction accuracies of two different feature selection approaches (outside of CV and within CV) for SVM and RLDA (unit: %) with respect to the number of selected features for the simulation data. Note that features were sequentially selected with higher votes, and the corresponding features were independently used to estimate prediction accuracies for each feature number. As the number of features selected by the within CV feature selection method varied in each CV loop, we averaged the prediction accuracies of a specific number of features selected in each CV loop. However, features fixed after the feature selection performed outside of CV were used to estimate prediction accuracies as a function of the number of features.

Figure 2 shows the changes in prediction accuracies with respect to the number of features from 1 to 40 when using the simulation data, showing the general trend of prediction accuracies as a function of the number of features. The prediction accuracy of the feature selection method performed within CV showed an increasing trend as the number of features increased until a certain number of features (about 10 for each classifier), but after which it showed a continuously decreasing trend. This phenomenon was well documented in the ML field, called the curse of dimensionality^15,16^. On the other hand, the prediction accuracy of the feature selection method performed outside of CV also showed an increasing trend with a small number of features, but after which it was saturated while retaining its accuracy similarly.

### Classification performance for actual clinical EEG data

For the feature selection method performed outside of CV, classification accuracies were over 70% for both classifiers, regardless of the number of selected features, and the mean classification accuracies of SVM and RLDA were 77.09 and 79.84%, respectively. On the other hand, for the feature selection method performed within CV, classification accuracies were less than 70% for both classifiers, and the mean classification accuracies of SVM and RLDA were 68.02 and 68.29%, respectively. The overall classification accuracy of the feature selection method performed outside of CV was higher than that of the feature selection method performed within CV by about 10% due to overfitting issue, regardless of the number of features (Table 2).

**Table 2:** Classification accuracies of the actual EEG data.

Vote threshold	Outside of CV			Within CV		
	n	SVM	RLDA	n	SVM	RLDA
≥ 50	54	77.65	79.06	37–67	69.10	68.65
≥ 60	45	77.76	81.91	30–53	67.69	69.18
≥ 70	39	77.53	78.92	23–41	69.13	68.20
≥ 80	31	75.97	79.79	15–32	67.58	68.25
≥ 90	19	76.56	79.54	9–21	66.61	67.16
Mean (S.D.)		77.09 (0.71)	79.84 (1.08)		68.02 (0.97)	68.29 (0.66)

Two feature selection approaches (outside of CV and within CV) were tested using two different classifiers (SVM and RLDA, unit: %) with respect to the different vote thresholds (number of selected features).

n* number of selected features, *S.D* standard deviation, *SVM* support vector machine, *RLDA* regularized linear discriminant analysis.

**Figure 3:**
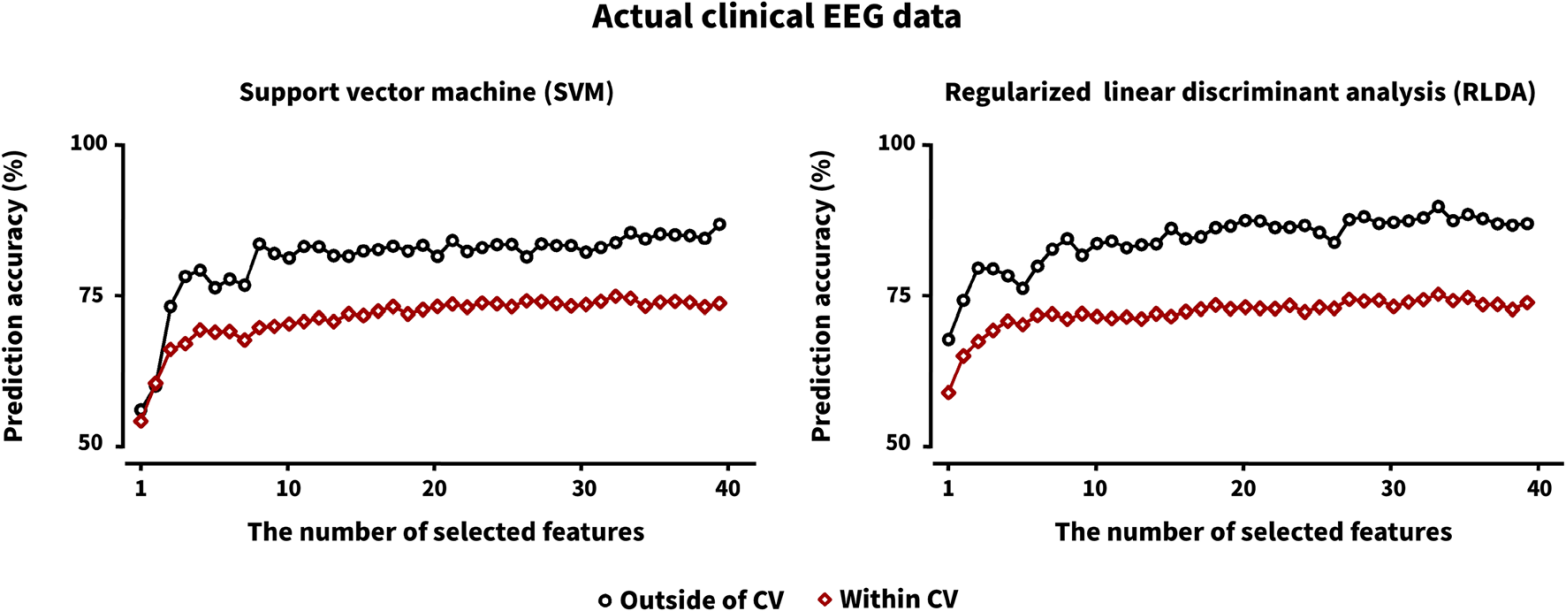
Prediction accuracies of two different feature selection approaches (outside of CV and within CV) for SVM and RLDA (unit: %) with respect to the number of selected features for the actual clinical EEG data. Note that features were sequentially selected with higher votes, and the corresponding features were independently used to estimate classification accuracies for each feature number. As the number of features selected by the within CV feature selection method varied in each CV loop, we averaged the prediction accuracies of a specific number of features selected in each CV loop. However, features fixed after the feature selection performed outside of CV were used to estimate classification accuracies as a function of the number of features.

Figure 3 represents the trend of classification accuracies when using the actual clinical EEG data with respect to the number of features from 1 to 40. Contrary to the results of the simulation data, a similar trend of classification accuracies was observed for the both feature selection methods in terms of the number of features, but with the difference of about 10% classification accuracy between the two feature selection approaches. The classification accuracy increased until a certain number of features (about 10 for each classifier), but after which it was saturated. In particular, when using the within CV feature selection method, the trend of classification accuracies obtained using the simulation data was different from that of those obtained using the actual EEG data. This difference would come from the different characteristics between the two datasets. In the case of the simulation data, data distribution was artificially controlled, and thus discriminable and non-discriminable features were more clearly divided, as compared to the actual clinical EEG data. Therefore, it seemed that uninformative features were added after the certain number of features (about 10 for each classifier) was selected in terms of the discrimination of two groups, which rather hindered the classification instead of helping the classification. On the other hand, in the case of the actual clinical EEG data, the features selected after the certain number of features (about 10 for each classifier) did not help increase the classification accuracy, and also did not hinder classification at least.

### Spatial distribution and overlap ratio of EEG features

**Figure 4:**
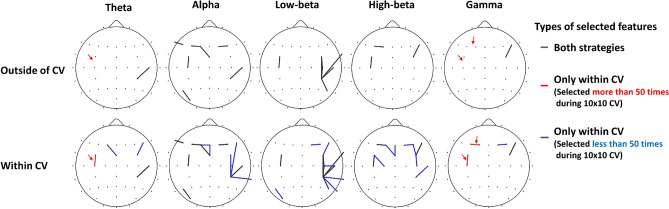
Spatial distribution of selected EEG features extracted from clinical EEG data when a vote threshold was 90 for the two feature selection approaches (outside of CV vs. within CV). Upper row and bottom row represent the spatial distributions of features selected by the outside of CV method and within CV method, respectively. Black lines represent the same features selected by the both feature selection approaches. Red and blue lines indicate the features selected by the within CV method more than and less than 50 times during 10 × 10 CV, respectively, but not selected by the outside of CV method at all.

Figure 4 shows the representative spatial distributions of selected EEG features extracted from the clinical EEG data for each feature selection strategy (outside of CV vs. within CV) when a vote threshold was 90. In the case of feature selection performed outside of CV, 19 features fixed before CV were used for classifying two groups (upper row). In contrast, in the case of feature selection performed within CV, more features were selected because different features were selected in each CV step. All features selected by the outside of CV method were overlapped with those selected by the within CV method (black lines), but some important features were not captured by the outside of CV method (red and blue lines in bottom row). Particularly, the three features selected by the within CV (one theta connection and two gamma connections, denoted by a red arrow) were not selected by the outside of CV method even though they were selected by the within CV method more than 50 times during 10 × 10 CV. Note that the three features were closely associated with the patients’ neurophysiological traits from the clinical point of view; PTSD patients generally showed the abnormal functional connectivity in frontal areas for theta and gamma frequency bands, and they were closely related to patients’ clinical symptoms, such as rumination and re-experiences^17–20^. Based on the result, it can be reasonably thought that some crucial features, which should be used to train a precise classification model, were discarded by the feature selection performed outside of CV.

**Figure 5:**
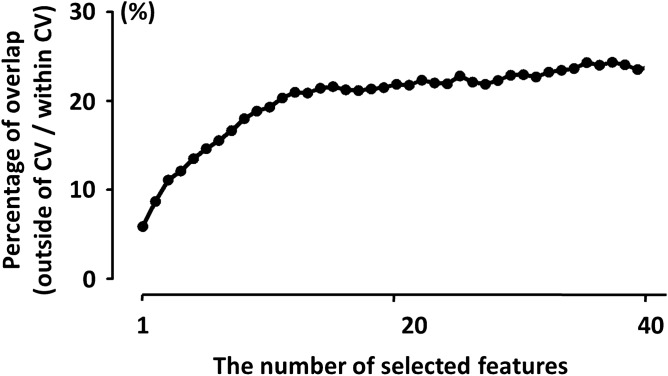
Mean overlap ratios (unit: %) between two feature selection approaches as a function of the number of selected features.

Figure 5 represents the average overlap ratios with respect to the number of selected features. The overlap ratio was kept under the 30% and it was saturated after about 10 features were selected, meaning that features selected by the two feature selection approaches were significantly different.

## Conclusion

The differences between the prediction accuracies of the two feature selection approaches can be thought of as the amount of overfitting due to selection bias. However, our results do not mean that those of the previous study^6^ using the feature selection method performed outside of CV were necessarily biased, but indicate that estimating prediction performance should not be performed after feature selection independently because prediction performance can be potentially biased, as shown in both Figs. 2 and 3. In other words, all steps in using ML, including feature selection, should be conducted within CV by separating training and test data to obtain an unbiased estimate of the true performance^11,12^.
